# Second Stage Caesarean Section and Subsequent Preterm Birth

**DOI:** 10.1111/ajo.70193

**Published:** 2026-11-02

**Authors:** Keyi Lau, Vanessa El‐Achi, Jenny M. Yang

**Affiliations:** ^1^ South West Sydney Clinical School University of New South Wales Liverpool New South Wales Australia; ^2^ Department of Obstetrics and Gynaecology Liverpool Hospital Liverpool New South Wales Australia

**Keywords:** caesarean delivery, preterm birth, second stage

## Abstract

Caesarean section during the second stage of labour has been associated with an increased risk of subsequent preterm birth. This risk has been hypothesised to be a product of inadvertent cervical trauma, compromising the integrity of the cervix in the subsequent pregnancy. Understanding the biological complications of this procedure may help guide clinician decision‐making to reduce future risk of preterm birth within this population.

**Abbreviations**
OVD: operative vaginal delivery
PTB: preterm birth
SSCS: second stage caesarean section
TAC: transabdominal cerclage
TVC: transcervical cerclage

## 1 Introduction

The rising incidence of Caesarean sections performed during the second stage of labour has become an area of increasing clinical concern, as it carries an increased risk of preterm birth (PTB) in subsequent pregnancies [1]. Driving factors may include decreasing experience and increasing reluctance of medical practitioners with mid‐cavity or rotational instrumental deliveries, increased maternal concern of potential pelvic floor injury following operative vaginal delivery, and late detection of malpresentations unsuitable for vaginal delivery [2, 3]. Within Australia, it has been reported that up to 60% of second‐stage Caesarean sections (SSCS) have been undertaken without a prior attempt at operative vaginal delivery (OVD) [4].

## 2 Methods

The databases PubMed, Medline, Scopus and Embase were used in the literature search. The search phrases ‘second stage caesarean section’, ‘subsequent preterm birth’, alongside secondary searches incorporating ‘hysterotomy extension’, ‘caesarean scar niche’ and ‘cervical cerclage’ and other search phrases were employed. In addition to the database searches, manual reviews of reference lists were conducted to ensure that all relevant studies were included. Our review did not include abstracts or conference articles. The articles were considered if their publishing date was from 2010 onwards, and only those written in English were considered.

## 3 Incidence of Subsequent Preterm Birth Following Second Stage Caesarean Sections

There is now growing evidence associating SSCS as an independent risk factor for subsequent PTB, with studies reporting increased risks of 1‐ to 5‐fold [5, 6, 7]. Levine et al., initially reported the risk of a subsequent PTB being 6.6 fold higher in women with a previous SSCS, compared to women with a CS in their first stage of labour (13.5% vs. 2.3%, *p* = 0.03) [8]. O'Sullivan et al. [9] also summarised the evidence associating spontaneous PTBs after SSCS, reporting odds ratio of 1.44 to 3.42 when compared with vaginal delivery. Women with a previous SSCS also face a 1.5 fold risk of PTB < 37 weeks' gestation and a 2 fold risk of PTB < 32 weeks' gestation, when compared to women with a previous spontaneous vaginal birth [5]. The hypothesis that changes in cervical stroma occurring with complete dilation increase susceptibility to premature cervical dilation in a subsequent pregnancy [8], is supported by a dose‐dependent response between cervical dilatation and subsequent PTB risk, with Wood et al.'s [5] reporting an increased risk of PTB following Caesarean section at 9–10 cm dilation, compared to emergency Caesarean sections performed when the cervix was dilated between 0 to 4 cm. However, it remains unclear whether this dose–response reflects direct cervical trauma or acts as a surrogate marker for duration and complexity of labour, which are themselves potential confounders. These studies have been further limited in their ability to isolate this association from other known confounders of spontaneous PTB. To address this, Sadler et al.'s and Woolner et al.'s [6, 7] studies specifically examined the risk of spontaneous PTB with increasing cervical dilatation, controlling for potential confounders. Sadler et al. [7] reported that subsequent PTB increased with increasing cervical dilatation > 4 cm in labour, with the highest rate of subsequent PTB found at full dilatation. Woolner et al.'s [6] study similarly found that a previous SSCS was associated with a greater risk of subsequent PTB compared with all other modes of first birth, particularly vaginal birth. However, studies have not established causation between SSCS and subsequent PTB, as indication bias remains a limitation, where clinical circumstances leading to SSCS (e.g., failed operative vaginal delivery) could influence subsequent PTB risk, independent of the SSCS itself.

## 4 Proposed Mechanisms

### 4.1 Hysterotomy Near the Internal Cervical Os

Recent evidence suggests the presence of a specialised ‘cervical sphincter’ at the internal os, comprised of 50%–60% of smooth muscle cells surrounding the endocervical canal [10]. Following cervical effacement to reach full dilatation, it is hypothesised that these smooth muscle cells could migrate into the lower uterine segment, inadvertently being incised during SSCS thus compromising the recovery of the cervical sphincter [11]. Furthermore, mucus‐producing cells in the endocervical mucosa could cause insufficient wound healing and niche formation if cervical tissue were incised, which could also compromise the integrity of the cervix [12]. Such factors may account for the increased risk of PTB associated with a lower hysterotomy, where a prospective cohort study by Banerjee et al. [13] found that mid‐gestation identification of a CS scar located within 5 mm of the internal cervical os was associated with an increased risk for cervical shortening and PTB in subsequent pregnancies, when compared to a CS scar of more than 5 mm above the internal cervical os.

The likelihood of a low hysterotomy appears to be increased with a CS performed close to or at full dilatation. In a prospective study by Kamel et al. assessing the location of the CS scar in postpartum women following a primary CS at term, it was found that the hysterotomy of women who underwent an emCS at ≥ 8 cm dilation was positioned at, or below, the internal cervical os in 97.7% of cases [14]. Advanced labour CS (cervical dilatation of 8–10 cm) was further associated with an 8‐fold risk of cervical scar location at or below the internal os, compared to early labour CS (cervical dilatation of 4–7 cm) [15]. Furthermore, Ivan et al. found that for each centimetre in cervical dilatation at the time of CS, the scar is positioned 0.88 mm more caudally in the uterus or cervix, with cervical dilatation demonstrating independent predictive power for low CS scar position [15].

### 4.2 Niche Formation

CS scars located at or below the internal cervical os are associated with an increased risk of suboptimal healing, such as developing a niche formation, an iatrogenic pouch‐like defect at the site of a previous CS scar due to defective tissue healing [16], compared to scars within the lower uterine segment [15]. Niche development is associated with an increased risk of gynaecological morbidity and serious obstetric complications in future pregnancies, such as abnormal placentation resulting in placenta accreta spectrum [17]. However, high‐quality prospective evidence directly exploring the impact of niche formation on subsequent PTB remains limited, with proposed mechanisms largely based on biological plausibility rather than controlled study data [18]. The prospective study by Ivan et al. [19] which assessed the CS scar characteristics of singleton pregnancy participants with a history of SSCS, found that CS scars positioned at or inferior to the internal cervical os demonstrated a higher likelihood of developing a niche with larger dimensions of those niches developed, compared to scars situated superior to the internal os (58% vs. 40%, *p* = 0.029). Osser et al. [20] identified that the risk of ‘large niche’ development, defined as a CS scar defect with an overlying myometrial thickness of ≤ 2.2 mm, increased if the CS was carried out at a cervical dilatation ≥ 5 cm. As mentioned earlier, this has been hypothesised to be due to the inclusion of cervical tissue within the suturing line, where the mucus‐producing endocervical mucosa leads to insufficient wound healing and thus increased niche formation [12]. In relation to spontaneous PTB, a proposed mechanism is that the presence of chronic inflammation in niches [21] could cause cervical length (CL) shortening in subsequent pregnancies [19]. Although the development of niches also depends on various patient‐ and surgery‐related factors, such as advanced maternal age ≥ 40, raised body mass index ≥ 25 and the use of locking sutures of both uterine layers [15], the above evidence suggests that SSCS is a preventable risk factor for niche formation and subsequent PTB risk.

### 4.3 Hysterotomy Extensions

SSCS is consistently identified as the strongest risk factor for hysterotomy extensions [22], which are also independently associated with an increased risk of PTB. Muraca et al. [23] found a 2.5‐fold higher risk of subsequent PTB < 34 weeks' gestation, compared to women without hysterotomy extensions. There is anatomical continuity of the effaced cervical tissue with the lower segment of the uterus during the second stage of labour, which is hypothesised to account for the increased susceptibility of the lower uterine segment to hysterotomy extensions, in addition to increasing the risk of inadvertent cervical incision as described above [22]. A low fetal head results in less space between the fetal head and the maternal pubic symphysis, which can cause difficulties in disimpaction, also increasing the risk of hysterotomy extensions [22, 24]. Erikkson et al. found that Caesarean deliveries at a low fetal station, defined as at or below the ischial spines, had a 1.73 increased odds of PTB < 32 weeks' gestation and 1.61 increased odds of spontaneous PTB, compared to Caesarean deliveries at a high fetal station [25]. A schematic diagram of the contributing mechanisms for subsequent PTB in women with a history of SSCS is shown in Figure 1.

**FIGURE 1:**
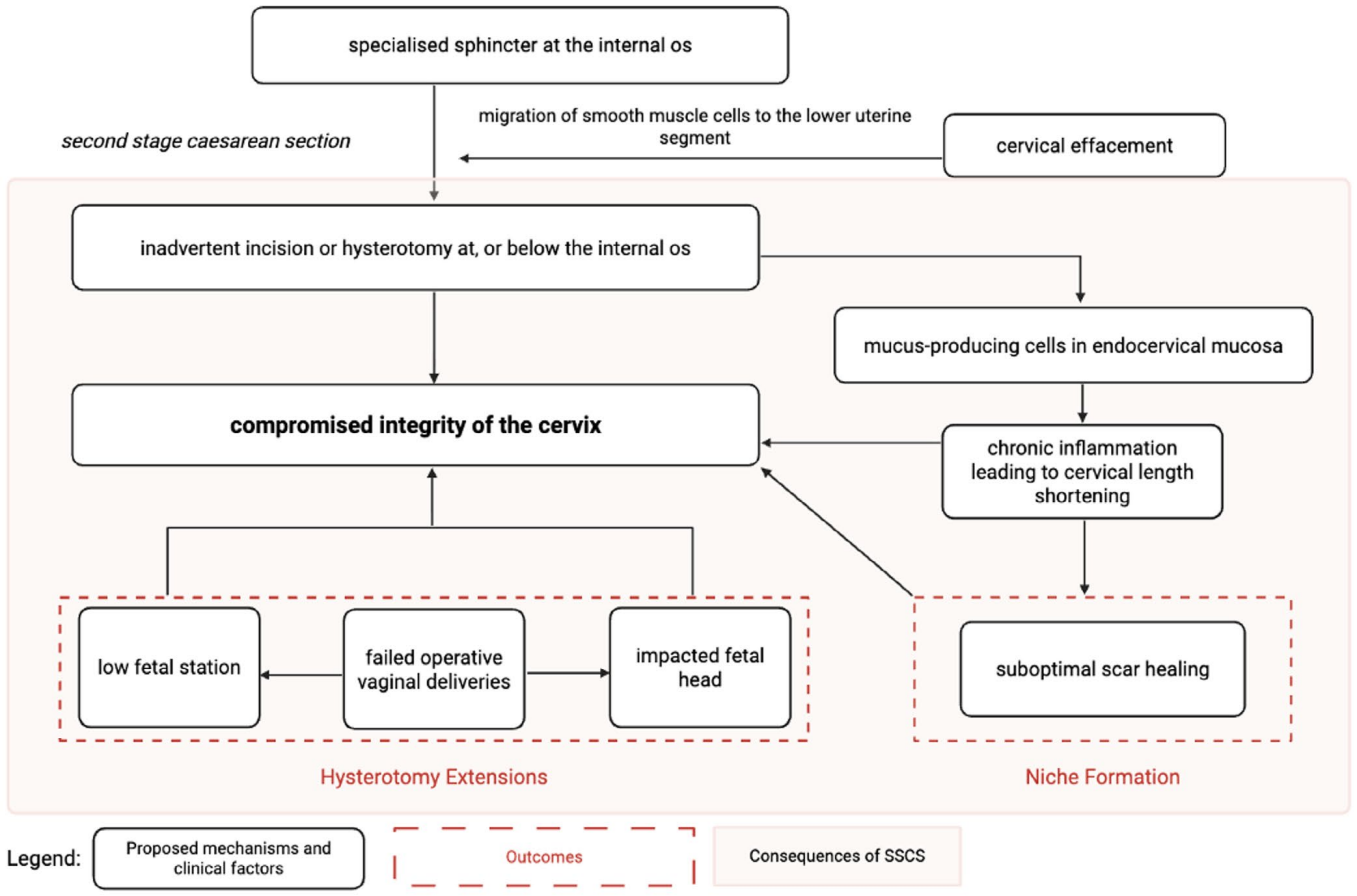
A schematic representation of the proposed mechanisms of cervical insufficiency following second stage caesarean section. Created with BioRender.com.

## 5 Measures to Reduce Risk of Subsequent Preterm Birth Following Second Stage Caesarean Section

PTB risk associated with SSCS may be targeted using strategies to reduce hysterotomy extensions associated with the impacted fetal head. With regard to disimpaction techniques, Cornthwaite et al. [26] summarised several systematic reviews which determined that ‘pull’ methods, such as reverse breech extractions, may be safer for women, as vaginal disimpaction was associated with an increased risk of uterine incision extensions. Furthermore, there have been recent findings that vaginal disimpaction results in a non‐statistically significant trend towards an increased risk of subsequent PTB, compared to reverse breech extraction [27]. However, larger prospective studies are required to assess this association and current findings are also limited by a lack of standardised reporting on surgeon experience and training, potentially resulting in selection bias. ‘Pull’ methods may also require an inverted T or J incision to improve access for the delivery of an impacted fetal head, which increases risk of uterine rupture in future pregnancies [28]. However, studies examining the impact of hysterotomy extensions on subsequent PTB have largely excluded patients with a previous inverted T or J incision, which may hence inappropriately skew the data in favour of ‘Pull’ methods. PTB risk following low hysterotomies and the development of a CS scar niche also requires further research exploring methods to facilitate optimal hysterotomy placement at the time of SSCS, ideally at least 5 mm superior to the internal cervical os, especially given the distortion of normal uterine anatomy at full dilatation.

Another target to reduce SSCS‐associated PTB risk is to prevent the index SSCS. Successful OVDs and a prolonged second stage of labour do not increase the risk of subsequent PTBs [29, 30]. In fact, Wang et al. [31] identified the absolute risk of PTB and the risk of spontaneous PTB in a subsequent pregnancy to be reduced with mid‐cavity OVD compared to SSCS (3.2% vs. 5.7%, *p* = 0.035) and (2.0% vs. 4.3%, *p* = 0.019) respectively. This study also attempted to reduce confounding factors by focusing on primiparous women with spontaneous PTB not related to the previous mode of delivery, such as fetal anomaly, antepartum haemorrhage or maternal medical disease with comparable factors at the time of delivery. However, in an observational study of pregnancies which reached complete dilatation, a prolonged second stage of labour was found to decrease the likelihood of vaginal delivery [32]. It must also be considered that a SSCS following a failed OVD attempt, where the fetal head was impacted in the maternal pelvis, has more than a two‐fold increased prevalence of subsequent PTB compared with SSCSs for other indications (8.2% vs. 3.8%, *p* < 0.001) [33]. This highlights the need to determine the likelihood of successful OVD more accurately before initiating an attempt. The discussion surrounding failed OVDs and increasing SSCS rates is a multifaceted issue to address in the current climate of increased maternal awareness of OVD‐associated pelvic floor injury [34], in conjunction with reduced obstetric training and confidence in performing complex OVDs [35]. This also highlights the importance of comprehensive antenatal education to facilitate informed maternal decision‐making intrapartum, ensuring that women are well‐prepared to weigh the potential risks and benefits, and enabling choices which align with their values and clinical circumstances. To increase successful OVD rates and reduce the incidence of SSCS, it is also important to focus on training and education to increase competence and confidence in performing OVDs when appropriate [34]. This was supported by a qualitative study which reported that the presence of senior obstetricians during the birth improved perceived team confidence and safety during the OVD [36].

To identify women at an increased risk of PTB following SSCS, transvaginal ultrasound assessment of the CS scar position relative to the internal cervical os—using the uterine arteries in the paracervical region as a landmark [16, 36]—should be considered, as scar position predicts subsequent cervical length shortening and spontaneous PTB [16]. This may facilitate early detection of cervical shortening and timely interventions to reduce PTB risk. Banerjee et al. [37] demonstrated the reproducibility of a midtrimester transvaginal ultrasound protocol for assessing the position of the hysterotomy scar in pregnant women with a previous SSCS, which allowed for the visualisation of hysterotomies in over 80% of women, offering a feasible tool to stratify the risk of subsequent PTB in clinical practice. Emerging research has also shown niche parameters on ultrasound to be significantly associated with the prediction of CL shortening ≤ 25 mm [13, 19], so future research is required to identify whether niche parameters can be an additional predictor for stratifying the risk of spontaneous PTB. The inflammatory mechanism behind niche formation has led some clinicians to propose that surgical repair of caesarean scar prior to subsequent pregnancy could potentially reduce PTB risk by eliminating the nidus of chronic inflammation [38]. However, current evidence for niche surgery in the prevention of PTB as an outcome is limited. Established indications for niche repair remain symptomatic relief [39] and improving fertility outcomes in women with residual myometrial thickness less than 3 mm [40], and further research is needed before niche surgery can be considered a PTB prevention strategy.

The inadvertent incision of hysterotomies and hysterotomy extensions within the cervical stroma during SSCs may account for the reduced efficacy of transvaginal cervical cerclages (TVC) in preventing PTBs in women with a previous SSCSs [41], although the exact mechanism of this finding requires more research. In keeping with this theory, there is some evidence that transabdominal cerclage (TAC) is more effective than TVC in preventing recurrent PTB in women with a history of SSCS and subsequent spontaneous PTB or late miscarriage, followed by at least one further pregnancy with cerclage [42]. This leads to the possibility that localisation of the hysterotomy scar may identify a subgroup of women for whom TAC may be worth considering should the hysterotomy be identified within the upper cervix, for whom TVC is less likely to be effective. Given that TACs are currently infrequently performed and require specific training, a more invasive open or laparoscopic approach to insertion, and an additional surgery for removal, further research is needed to validate whether TACs are superior in preventing PTBs in this population and hence justify increased resource allocation and training for this procedure. Of note, the ongoing randomised controlled ABOVE trial, comparing the effectiveness of TACs to TVCs in women who have experienced a late miscarriage or spontaneous PTB following a SSCS [43], will continue to build the evidence base for the use of TAC versus TVC as the primary procedure in this population.

A schematic diagram of the above‐mentioned factors for managing second‐stage Caesarean sections is presented in Figure 2.

**FIGURE 2:**
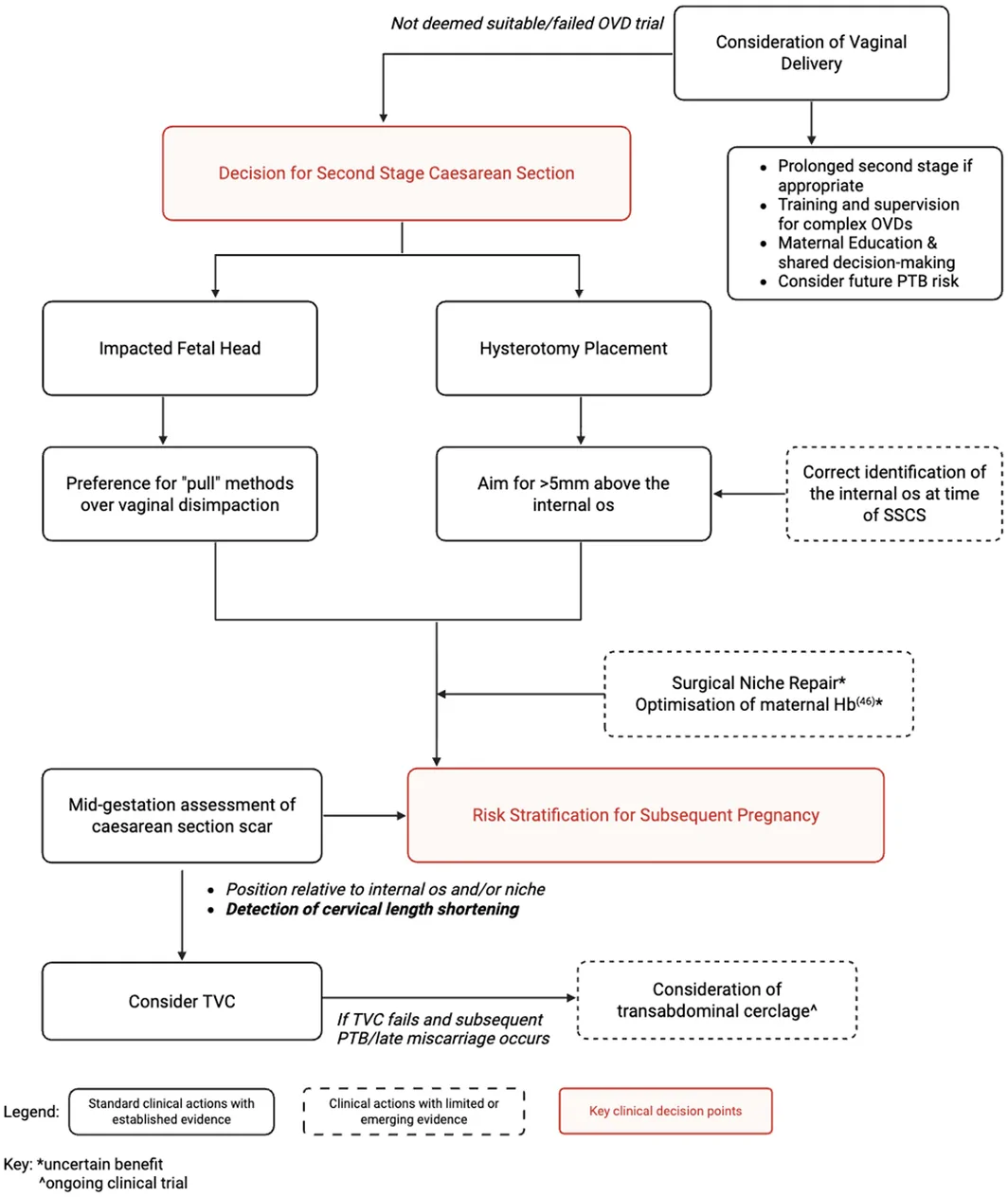
A schematic representation of the suggested management of a second‐stage caesarean section, including intraoperative considerations and postpartum risk stratification for preterm birth. Created with BioRender.com.

## 6 Conclusion

SSCSs are associated with subsequent PTB. Current evidence for the biological mechanisms underlying this association is limited and highlights the importance of further research in this area to identify primary and secondary prevention strategies to the reduce risk of subsequent PTB.

## Conflicts of Interest

The authors declare no conflicts of interest.

## Data Availability

Data sharing not applicable to this article as no datasets were generated or analysed during the current study.
